# Bimodal Cochlear Implants: Measurement of the Localization Performance as a Function of Device Latency Difference

**DOI:** 10.1177/23312165251396658

**Published:** 2025-11-24

**Authors:** Rebecca C. Felsheim, Sabine Hochmuth, Alina Kleinow, Andreas Radeloff, Mathias Dietz

**Affiliations:** 1 Department für Medizinische Physik und Akustik, 597451 Carl von Ossietzky Universität Oldenburg, Oldenburg, Germany; 2 Cluster of Excellence 597459 “Hearing4All”, Oldenburg, Germany; 3 Department of Otolaryngology, Head and Neck Surgery, 11233 Carl von Ossietzky Universität Oldenburg, Oldenburg, Germany; 4 Research Center Neurosensory Science, University of Oldenburg, Carl von Ossietzky Universität Oldenburg, Oldenburg, Germany

**Keywords:** bimodal hearing, sound localization, interaural latency mismatch, cochlear implant, hearing aid

## Abstract

Bimodal cochlear implant users show poor localization performance. One reason for this is a difference in the processing latency between the hearing aid and the cochlear implant side. It has been shown that reducing this latency difference acutely improves the localization performance of bimodal cochlear implant users. However, due to the frequency dependency of both the device latencies and the acoustic hearing ear, current frequency-independent latency adjustments cannot fully compensate for the differences, leaving open which latency adjustment is best. We therefore measured the localization performance of 11 bimodal cochlear implant users for multiple cochlear implant latencies. We confirmed previous studies that adjusting the interaural latency improves localization in most of our subjects. However, the latency that leads to the best localization performance for most subjects was not necessarily at the latency estimated to compensate for the interaural difference at intermediate frequencies (1 kHz). Nine of 11 subjects localized best with a cochlear implant latency that was slightly shorter than the estimated latency compensation.

## Introduction

Binaural hearing, that is, hearing with two ears, enables listeners to pinpoint the direction of a sound with remarkable accuracy. Trained normal hearing listeners can distinguish directions of two sources separated by angles as small as 1° in front of the head (Mills, 1958). In listeners with impaired hearing, the ability to localize sounds is reduced compared to normal hearing listeners. In a study by Dorman et al. (2016), which compared the localization performance of different subject groups, bilateral hearing aid users had an average root mean square error (RMSE) of 12° and bilateral cochlear implant (CI) users had an average RMSE of 29°. This stands against an average RMSE of 5° in an approximately age-matched normal hearing control group. An even larger average RMSE of 62°, close to chance performance, was reported in a small group of bimodal CI users with a hearing aid contralateral to the CI.

Most bimodal CI users used to be severely hearing impaired in both ears but were only eligible to receive one CI due to limited coverage by health care. With a high-power hearing aid, these patients receive some low-frequency hearing to complement their dominant CI, but, not surprisingly, they perform very poorly in sound localization, with little to no spectral overlap between CI and hearing aid stimulation. The bimodal participants in Dorman et al. (2016) appear to fall into this category. However, when health care coverage is less restrictive, a second very different group of bimodal CI users can be found: Those who have only a moderate hearing loss in one ear, that is best treated with a hearing aid, and a severe-to-profound hearing loss in the other ear, treated with a CI. These patients can already understand speech with their hearing aid alone and opt for the CI to get a more symmetric hearing sensation as well as to restore their spatial hearing. While better spatial hearing should be possible in this group due to a spectral overlap of both stimulation modalities, even this group has a rather poor sound localization with an average RMSE of 53° (Angermeier et al., 2021).

Multiple reasons are conceivable for the lower localization performance of the bimodal CI users. For instance, the human brain may not be able to integrate the two very different modalities of electrical and acoustic hearing well enough to enable binaural processing. Other possible causes are the mismatches between the two ears caused by the two different devices and differently impaired ears. These include tonotopic mismatch, level mismatch, latency mismatch, and a mismatch in the spectral content between the two ears, caused by the CI not being able to selectively stimulate the apical turn and high-frequency limitation in the acoustic hearing ear (see Pieper et al., 2022 for a review).

The level mismatch has its roots in the often-independent fitting of the two devices and the reduced dynamic range, especially in electric hearing. While it might be possible to balance the levels in the two devices for a specific stimulus, the reduced dynamic range makes it difficult to balance the levels for a wider range of stimuli. The independent, and possibly different, compression of the dynamic range also leads to different representation of amplitude modulation in the hearing aid and the CI.

The last dimension of mismatch is latency: In the acoustically stimulated ear, this latency consists first of the frequency and manufacturer-dependent processing latency of the hearing aid (1–7 ms; Zirn et al., 2015) and on the frequency and level-dependent latency of the human ear (1–8 ms; Ruggero & Temchin, 2007). For the CI side, only the latency of the device needs to be considered, which is frequency dependent and ranges from 0.5 to 7 ms for MED-EL CIs (Zirn et al., 2015), simulating the frequency dependency of the human ear, and a nearly frequency independent latency of about 12 ms for Cochlear devices (Engler et al., 2020). These different latencies add up to a latency mismatch of several milliseconds, vastly exceeding even the highest acoustically caused interaural time differences (ITDs) of about 740 µs (Hartmann & Macaulay, 2014), corresponding to a sound from 90°.

For bimodal MED-EL CI users, the latency on the CI side is lower than the combined latency (hearing aid latency + ear latency) on the acoustic side for all frequencies and hearing aid manufactures (Zirn et al., 2015). Therefore, adding an additional delay to the CI processor is a promising option that is included in the most recent versions of the clinical fitting software Maestro since 2020. At present, only a frequency independent delay can be added. When ear and device latencies are known or estimated, the presumed interaural latency mismatch can be derived. We call it “estimated latency mismatch,” and this value can be added to the CI processor.

Angermeier et al. (2021) measured the localization performance in bimodal MED-EL CI users with and without compensating for the estimated latency mismatch. They found that compensating for the estimated latency mismatch in bimodal patients acutely improved the localization performance significantly from an average RMSE of 53° to 38°. No further improvement was found after a familiarization period of 3 weeks (Angermeier et al., 2023).

As the MED-EL CI latency is very similar to the latency of an acoustically stimulated ear, the estimated latency mismatch is approximately equal to the hearing aid latency, especially at frequencies near 1 kHz. At lower frequencies, the CI is a bit slower, and at higher frequencies even a bit faster (Zirn et al., 2015). Angermeier et al. (2021) therefore tested three different compensation delays: One that is equal to the hearing aid latency, 1 ms more, and 1 ms less. For five of seven listeners, the lowest RMSE was obtained with the largest delay. Localization bias without added CI latency was always to the side of the CI and decreased as expected with increasing CI delay. But even for the largest delay, six of seven listeners had a residual bias towards the CI. Similarly, Seebacher et al. (2019) measured the localization performance in single-sided deaf patients, who had normal hearing on the ear contralateral to the CI, and found the best localization performance when delaying the CI by 1 ms. This unexpected longer latency may have been caused by mismatches in other dimensions, such as level (Pieper et al., 2022). We hypothesize that an even larger CI delay would compensate for the residual bias, possibly even further reducing the RMSE as well. A good compensation could possibly be able to reduce the RMSE to the range of bilateral CI users, as also hypothesized by Pieper et al. (2022). In this work, we have therefore measured the localization performance of 11 bimodal CI users with MED-EL CIs over a wider range of added CI latencies.

## Methods

### Estimated Latency Difference

Zirn et al. (2015) estimated the latency difference between the two ears based on measurements of the wave V of the auditory brainstem response. Following their approach, the latency on the acoustic hearing side is given as

(1) $$\begin{array}{c} \tau_{a}(f)=\tau_{\mathrm{HA}}(f)+\tau_{\mathrm{ear}}(f)+\tau_{V} \end{array}$$

with $\tau_{\mathrm{HA}}(f)$ being the potentially frequency-dependent latency of the hearing aid, $\tau_{\mathrm{ear}}(f)$ the frequency dependent-latency of the ear, and $\tau_{V}$ the frequency-independent latency of the neural processing until wave V occurs. Similarly, for the electrically hearing side, the latency can be obtained as

(2) $$\begin{array}{c} \tau_{e}(f)=\tau_{\mathrm{CI}}(f)+\tau_{\mathrm{add}}+\tau_{V} \end{array}$$

where $\tau_{\mathrm{CI}}(f)$ is the frequency-dependent latency of the CI and $\tau_{\mathrm{add}}$ an additional latency that can be added to the processing latency. The value $\tau_{V}$ is assumed to be the same for the acoustic and electric hearing side. The latency difference is then given as

(3) $$\begin{array}{c} \Delta \tau (f)=\tau_{a}(f)-\tau_{e}(f)=(\tau_{\mathrm{HA}}(f)+\tau_{\mathrm{ear}}(f))-(\tau_{\mathrm{CI}}(f)+\tau_{\mathrm{add}}) \end{array}$$

For MED-EL devices the difference between $\tau_{\mathrm{ear}}$ and $\tau_{\mathrm{CI}}$ is very small. At 500 Hz $\tau_{\mathrm{ear}}$ is marginally smaller, at 1 kHz they are virtually identical and at 2 and 4 kHz $\tau_{\mathrm{ear}}$ is even a bit larger (Zirn et al., 2015). However, currently the clinical MED-EL software only allows for a frequency-independent additional latency. Therefore, a frequency-independent version of Δτ(f) needs to be estimated. We follow the approach of Angermeier et al. (2021) and assume an average equality between $\tau_{\mathrm{ear}}$ and $\tau_{\mathrm{CI}}$ . This simplifies Equation 3 to

(4) $$\begin{array}{c} \Delta \hat{\tau }={\hat{\tau }}_{\mathrm{HA}}-\tau_{\mathrm{add}} \end{array}$$

as frequency-independent estimation of the latency difference between the two ears, with the frequency-independent estimate of the hearing aid latency ${\hat{\tau }}_{\mathrm{HA}}$ .

For most participants, the hearing aid latencies ( ${\hat{\tau }}_{\mathrm{HA}}$ ) were measured with the hearing aid analyzer unit ACAM 5 (Acousticon GmbH, Reinheim, Germany) using tone bursts in the frequency range of 500 to 4000 Hz. The hearing aid latency was measured from the time difference of the rising flank of the envelope between input and output. The median of the measured latencies in the frequency range of 500 to 4000 Hz was taken as the general hearing aid latency. Due to technical problems, the latency could not be measured in three subjects (S03, S04, and S06). Therefore, the hearing aid latency was taken from the device delay list provided by MED-EL^1^. The subject-specific hearing aid latencies are shown in Table 2. The hearing aid latency can be compensated for in the MED-EL CI fitting software Maestro starting from Version 9 for processors Sonnet 2 and Rondo 3 or newer.

### Participants

Participants were recruited from the clinical CI-center and all gave written informed consent. The study was approved by the local ethics committee (2020-022). The experiments were conducted in a sound-treated room at the CI-center.

**Figure 1.**
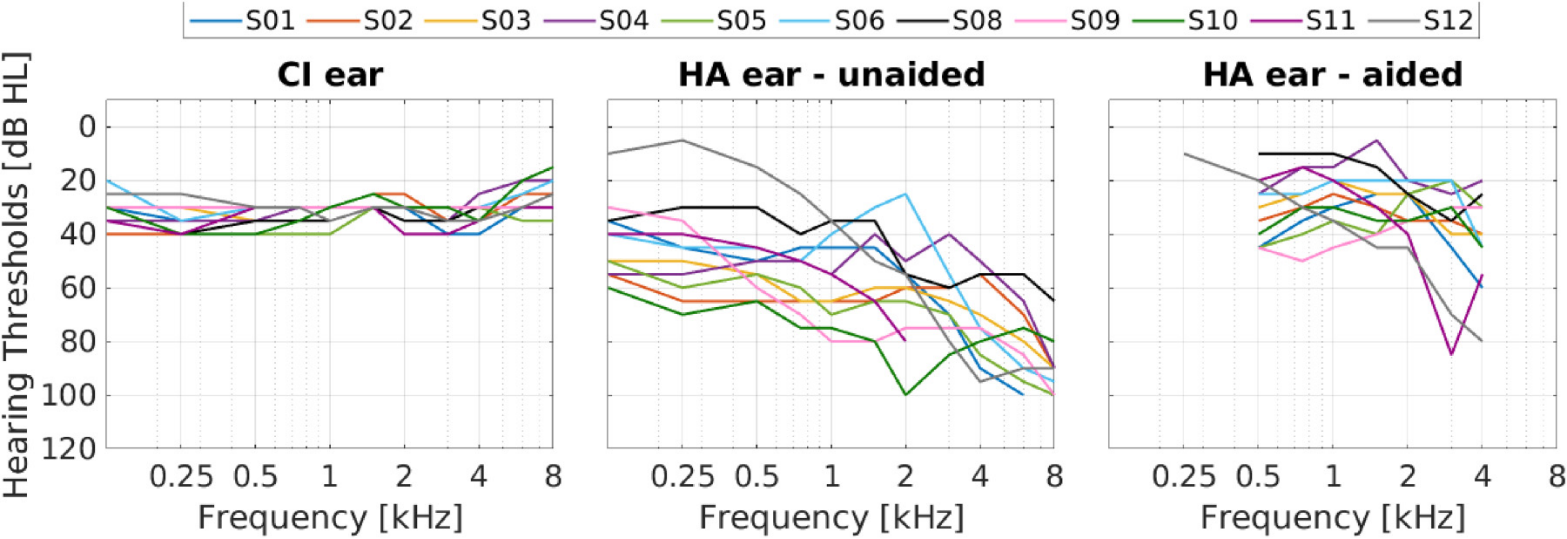
Individual Hearing Thresholds With the Cochlear Implant (CI, Left Panel), Unaided Pure-Tone Thresholds Contra-Laterally to the Implanted Ear (Middle Panel) and Aided Thresholds With the Hearing Aid (HA, Right Panel) of all Participants.

Eleven bimodal MED-EL CI listeners participated in the experiment. Their age ranged from 58 to 86 years. The listeners were fitted with a hearing aid contralaterally to the CI. Both devices were used daily. Unaided pure-tone averaged thresholds at 0.5, 1, 2, and 4 kHz (PTA4) on the hearing aid side ranged from 44 to 80 dB HL. Note that S11 had no measurable thresholds above 2 kHz. Therefore, a threshold of 120 dB HL was used for the PTA4 calculation at 4 kHz in this case. CI thresholds and both aided and unaided thresholds on the hearing aid side are shown in Figure 1.

Participants had at least four months of CI experience and achieved 50% or more correct responses with the Freiburg Monosyllables Test (speech perception in quiet) at a level of 65 dB SPL with each device, except for subject S02 and subject S09 who only achieved 25% and 35% correct with the hearing aid, respectively. Four CI users were already fitted with a hearing aid latency compensation $\tau_{\mathrm{add}}={\hat{\tau }}_{\mathrm{HA}}$ on their personal processor. An overview of the subject-specific information is given in Tables 1 and 2.

**Table 1.** Subject-Specific Data of Our Bimodal CI-Subjects.

ID	Age (years)	Personal Processor Type	Implant Type	De-Activated Electrodes	Duration of Implant Use (Years)
S01	58	Sonnet	Flex26	EL12	4.4
S02	70	Sonnet2	FlexSoft	None	0.4
S03	64	Sonnet	FlexSoft	None	6.2
S04	86	Sonnet2	FlexSoft	EL9-12	1.0
S05	66	Sonnet2	FlexSoft	EL11–12	8.9
S06	60	Sonnet2	FlexSoft	None	1.3
S08	60	Rondo3	FlexSoft	None	2.1
S09	69	Sonnet2	Flex24	None	6.8
S10	73	Sonnet2	FlexSoft	None	2.8
S11	59	Rondo3	FlexSoft	EL12	0.8
S12	70	Sonnet2	FlexSoft	none	1.0

**Table 2.** HA Latencies, Speech Perception Score, and the Unaided PTA4 of the Bimodal CI Subjects.

ID	Estimated HA Latency (ms)	Everyday HA Latency Compensation (ms)	Speech Perception in Quiet (% Correct)		Unaided PTA4 HA-Side (dB HL)
			CI	HA	
S01	8.2	None	70	65	60
S02	7.2	None	75	25	61
S03	5.2*	None	80	85	63
S04	9.3*	None	50	70	51
S05	8.3	None	65	65	69
S06	6.9*	None	80	100	46
S08	2.5	2.6	80	90	44
S09	8.3	None	75	35	73
S10	8.2	8.7	55	50	80
S11	6.3	6.3	65	90	75
S12	6.1	6.0	55	50	50

Estimated hearing aid (HA) latency was generally measured with a hearing aid analyzer unit (median latency in the frequency range between 500 and 4000 Hz). Latencies marked with an asterisk were taken from the ‘device delay list’ provided by MED-EL. The everyday hearing aid latency compensation refers to the programmed hearing aid latency used in the subjects’ personal CI processors. “None” indicates that no HA latency compensation was used. Speech perception in quiet was measured at 65 dB SPL with a monosyllabic word test (Freiburg Monosyllables test).

### Experimental Setup

Sound source localization measurements were performed in a sound-treated room. The measurement setup consisted of 13 loudspeakers (8030C studio monitors, Genelec, Finland) placed in a semicircle with a radius of 1.35 m at ear height and 15° apart in the frontal azimuthal half-plane. Each stimulus consisted of three 70-ms long Gaussian broadband white noise bursts, each gated with a 5-ms Hanning window, separated by two 30-ms long silent intervals (similar to Angermeier et al., 2021). Each localization measurement consisted of 55 stimulus presentations (5 times from each of the 11 most central loudspeakers). The presentations were randomized. Stimuli were presented at a level of 65 dB SPL. Random level roving of ±5 dB was applied. Calibration was performed with a hand-held level meter (PCE-322A, PCE-Deutschland GmbH, Germany) at the subject's head position.

All listeners used the same CI processor (MED-EL Sonnet2) during the measurements, which was programmed with the most frequently used individual map of each subject. All listeners, except for subject S04, used the FS4-processing strategy with four fine structure channels in their individual maps. Subject S04 used only three fine structure channels. During testing, the adaptive intelligence program was disabled, and the microphone setting was set to “natural.” On the acoustic side, the listeners used their individual hearing aid with their everyday settings. Subject S11 had a slightly louder sound impression with the CI processor used for testing than with the personal processor, a Rondo3. Therefore, the CI-volume was reduced one step in the volume settings, which corresponded to a volume reduction of 4% (about 18 current units). All other subjects performed the experiments at the average of their everyday CI volume setting.

The global hearing aid latency compensation setting in the CI $\tau_{\mathrm{add}}$ was systematically varied for each measurement using the MED-EL fitting software MAESTRO 9. In general, six different latencies between 1.5 and 10 ms were applied (1.5, 2/3, 4/4.5, 6, 8, and 10 ms). Note that the software requires a minimal CI delay setting of 1.5 ms. This is probably due to a longer processing latency of 1.5–2.0 ms in the Sonnet 2 compared to the Opus 2 (Zirn et al., 2025).^2^ Due to the lower limit of 1.5 ms enforced by MEASTRO, we assume that the minimum latency of the Sonnet 2 is 1.5 ms longer and we further assume that all extra latency values provided by MEASTRO are relative to the processing latency measured by Zirn et al. (2015). If the everyday hearing aid compensation setting of a subject was close to one of the experimental latencies mentioned above, the everyday hearing aid compensation setting was used instead (e.g., subject S11 performed the localization experiment with a latency of 6.3 ms instead of 6 ms). In the case of subject S10 an additional measurement with an additional latency of 12 ms was conducted, since the hearing aid latency measurement using the hearing aid analyzer unit suggested a substantially higher latency in the lower frequencies (median of 11.3 ms in the frequency range between 500 and 1000 Hz) compared to the higher frequencies (median of 8.1 ms). The first localization measurement was performed with the participants’ everyday hearing aid compensation setting (see Table 2 for individually used hearing aid compensation settings). The following localization measurements were conducted with randomized latency settings. In addition to the measurements with systematically varying latency settings, a retest measurement was performed at the end of the measurement session. Eight of the 11 subjects performed the retest, which was done with the same latency setting as used for the first measurement. Four listeners did not perform all six planned latency measurements and retest due to technical problems (S01), fatigue (S04, S09), and pure monaural stimulus perception (S12).

## Results

On average, the RMSE of the localization with the clinical settings was 39°, compared to 40° for Angermeier et al. (2023). When adjusting the latency settings to minimize the estimated latency mismatch, the mean RMSE improved marginally to 37°, compared to 32° for Angermeier et al. (2023). The mean of the lowest RMSE across all tested latencies was 32°.

**Figure 2.**
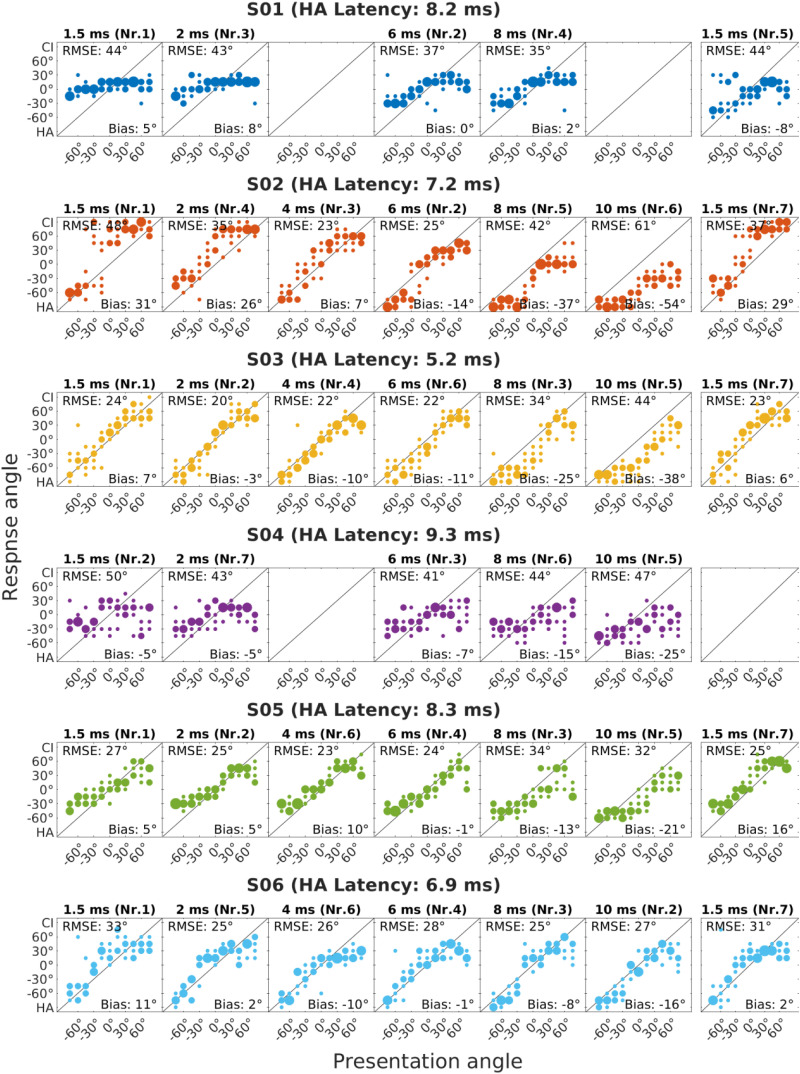

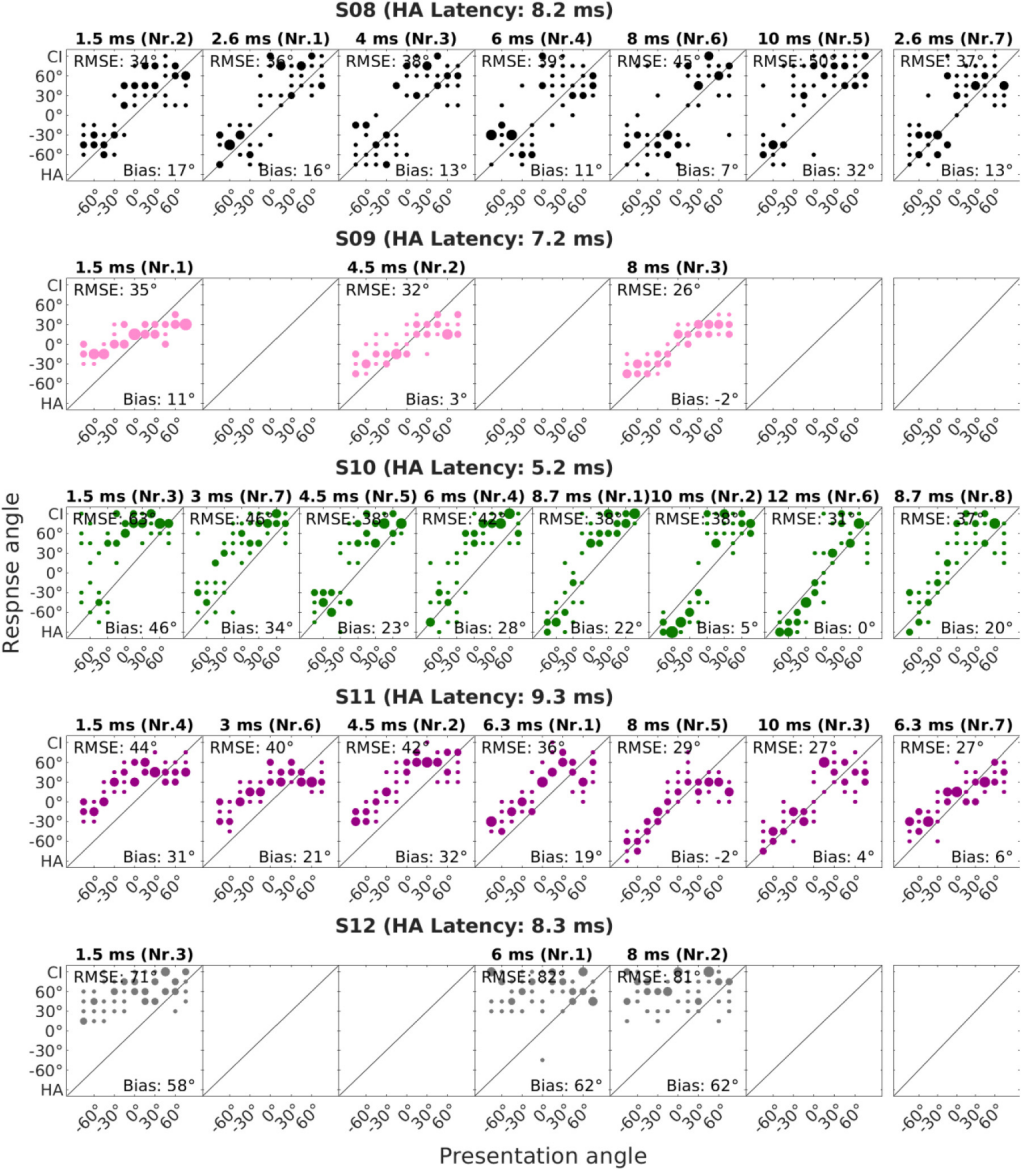
Bubble Plots Indicating the Response Angle Over Presentation Angle for Each Subject and Each of the Additional CI Latencies Measured. Data was Mirrored as Needed, Such That the Hearing Aid (HA) Side is Always at −90° and the CI side at +90°. the Overall Root Mean Square Error (RMSE) and Bias is Shown in Each Subplot at The Top Left and Bottom Right Corner, Respectively. The Number on Top of Each Plot Indicates the Measurement Order for the Respective Subject.

Figure 2 shows the responded angle (*y*-axis) over presentation angle (*x*-axis), for each subject and each of the latencies added in the CI settings. Negative angles refer to the side of the hearing aid and positive angles to the side of the CI. Each dot indicates a response, and larger dots are used to illustrate the same response for multiple repetitions of a presentation angle. If the active speaker was correctly identified the response would lie on the diagonal, which is illustrated by a line. At the top left of each panel, the RMSE for the current condition is given, and on the bottom right, the bias is depicted.

In cases with little or no added latency we expected a bias towards the CI. With increasing CI latency, we expected a shift in localization performance toward the center, combined with a decrease in RMSE, up to a subject-specific point. As latency was further increased, we expected an increasing bias towards the hearing aid side and again an increase in RMSE, since at some point the hearing aid side has a lower latency compared to the CI side. This pattern was observed in subjects S02, S03, S05, and S06. S03 had an overall lower bias compared to S02 and especially a lower bias towards the CI side. S10 and S11 showed a similar pattern, although with more noisy responses. Both S05 and S11 tended not to use the full range of possible responses.

Subject S01 tended to localize everything towards the midline with very few responses to either side. This became slightly less pronounced with increasing latency, but also with practice, as the retest at the end showed. Similar responses were shown by S04 and S09, with a very noisy response pattern for S04, which did not become much clearer as the added latency was increased.

In contrast to the patterns above, S08 localized everything more to the sides. In the bubble plots themselves, it is difficult to see the influence of added latency, but when looking at the RMSE and bias a systematic effect can be seen. Subject S12 was completely unable to perform the task, as every stimulus was recognized from the CI side, and even there the loudspeaker selection was apparently random.

**Figure 3.**
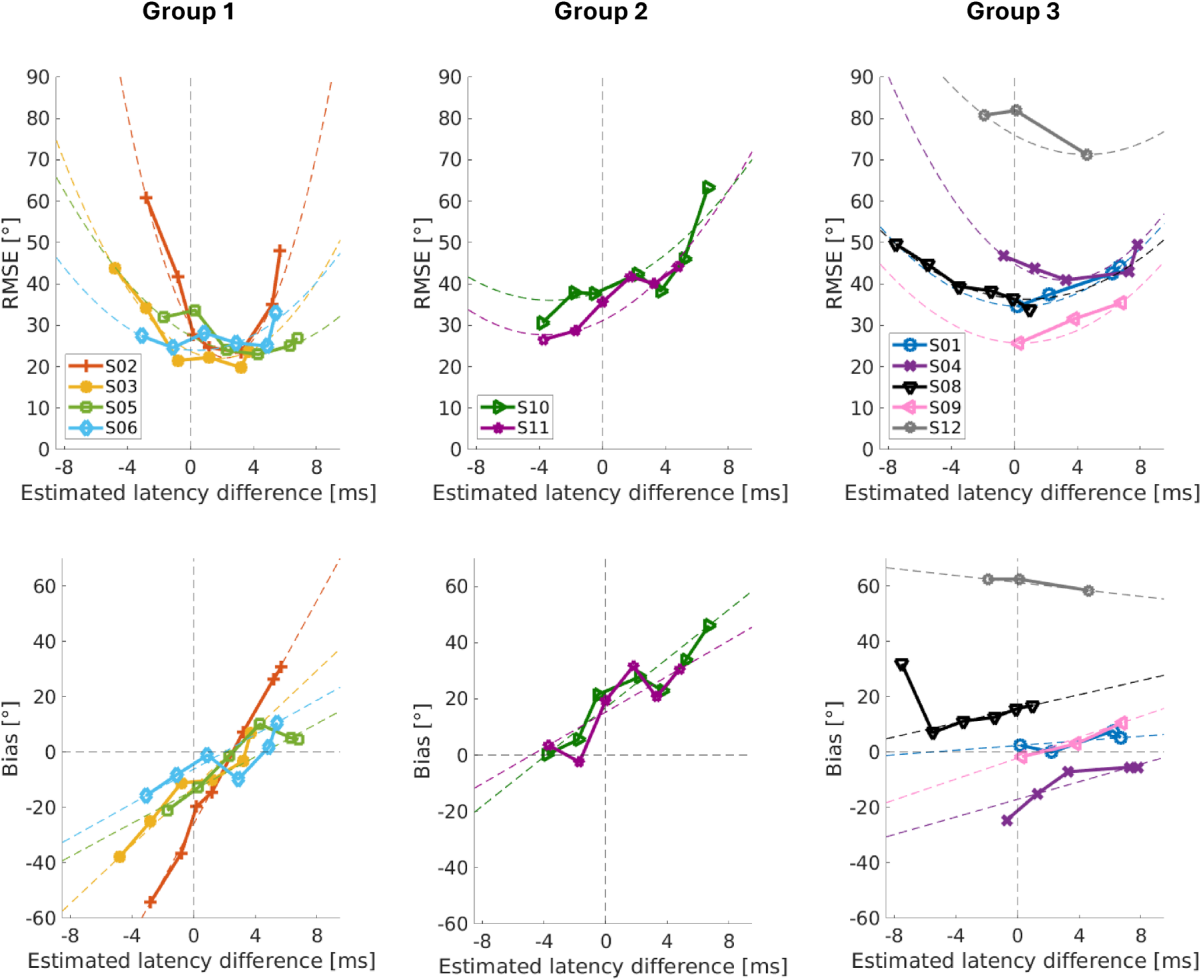
The Upper Row Shows the Root Mean Square Error (RMSE) Over the Estimated Latency Difference for the Three Subject Groups. A Positive Estimated Latency Difference Indicates a Faster CI, While a Negative Value Indicates a Faster Hearing Aid. Additionally to the Data, a Squared Fit for Each Subject is Shown With Dashed Lines. The Lower Row Shows the Bias Over Latency Difference for the Same Three Groups. The Bias Values Were Fitted With a Linear Function, Also Shown with Dashed Lines. The First Group Contains the Subjects That had the Best Latency Difference Slightly on the CI side. The Second Group Contains Those Subjects With the Best Latency on the Hearing Aid Side. Finally, the Third Group Contains the Subjects who had Trouble Performing the Task and Could Therefore not be Classified in Group 1 or 2.

The first step in the data analysis was to evaluate the RMSE and bias for each condition and subject over the estimated latency difference ( $\Delta \hat{\tau }$ , Figure 3), which we defined in Equation 4 as the averaged hearing aid latency minus the additional CI latency. A value of zero indicates an equalized latency between CI and hearing aid according to the estimated hearing aid latency. A negative estimated latency difference indicates that the hearing aid is faster than the CI, and thus the CI is faster for positive values. To reduce the influence of the measurement noise, Figure 3 also shows quadratic fits for the RMSE and linear fits for the bias in the dashed lines in addition to the measured values. For the quadratic fits, we constrained the *x*-axis shift to the range of the measured values.

We divided the subjects’ results into three groups based on their fitted RMSE and bias patterns and plotted them separately. Group 1 (first column in Figure 3) had the lowest fitted RMSE and the lowest fitted absolute bias for a positive estimated latency difference (or faster CI), implying that the estimated hearing aid latency was too long to achieve minimal RMSE and bias. This is the opposite to what Angermeier et al. (2021) reported. For all subjects in group 1, the RMSE showed the U-like shape we expected, with increasing error values as the estimated latency difference moves away from the optimum. This optimum, however, was not a clear minimal value, but multiple latency differences seemed to result in a similar error.

Group 2 (second column in Figure 3) had the lowest RMSE and absolute bias for negative estimated latency differences (or faster hearing aid), implying that the estimated hearing aid latency was too short to achieve minimal RMSE and bias, as in Angermeier et al. (2021). The RMSE of the subjects in group 2 just decreased with a higher added latency, and fitting a parabola in this data might seem a bit arbitrary. However, it is reasonable to assume that a further increase in the additional latency would cause the RMSE to increase again, as for group 1.

The bias values for both the groups 1 and 2 had a general tendency to move from the CI side (positive estimated difference) to the hearing aid side as the additional latency increased. For all subjects in these two groups, the bias was close to zero when the RMSE was minimal.

Finally, for subjects in group 3 (third column in Figure 3), there was either not enough data or the data was not clear enough to decide on one of the previous groups. This includes those subjects who had a strong tendency to localize either to the front or to one or both sides. Regardless of the location of the lowest RMSE and bias, it can be seen that the latency difference had a systematic influence on the localization performance as described by RMSE and bias for all subjects, except for S12, who was not able to localize at all. All subjects in group 3 had some problems with localization, which is reflected in the higher RMSE values. Nevertheless, the RMSE values changed systematically when the latency was changed, except for S12. This shows that even subjects with limited binaural fusion can benefit from adjusting the latency. However, the bias values in group 3 were also less clear, and for S08 and S12 a large bias towards the CI side remained even for large values of $\tau_{\mathrm{add}}$ .

**Figure 4.**
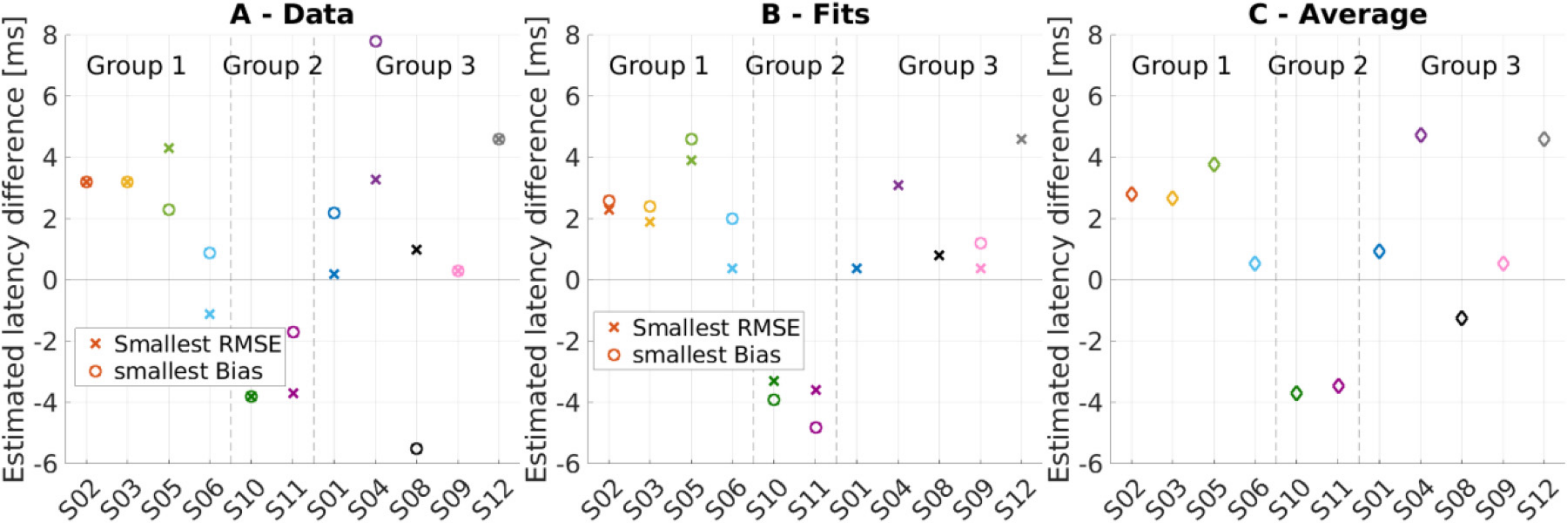
The Plot in Panel A Shows the Best Estimated Latency Difference Based on the Values Leading to the Lowest RMSE and the Lowest Bias, as Obtained the Measured Data. A Positive Estimated Latency Difference Indicates a Faster CI, While a Negative Value Indicates a Faster Hearing Aid. In Panel B, the Estimated Latency Difference of the Lowest RMSE and Bias is Shown, as Obtained From the Fits of the RMSE and Bias Data. Panel C Shows the Average Estimated Latency Difference of the Lowest RMSE and Bias of the Values Shown in Panels A and B.

Figure 4 compares the estimated latency differences resulting in the lowest RMSE and lowest bias in different ways. Panel A shows the estimated latency difference of the lowest measured RMSE and bias. For five of the 11 subjects, the estimated latency difference based on the lowest measured bias and the lowest measured RMSE led to the same results. For the other subjects, there seemed to be a gap between the two values. However, this may be partly due to the very discrete step size of 1.5 to 2 ms used in this experiment. Only for S04 and S08 the best estimated latency differences based on bias and RMSE were more than one step apart. Panel B in Figure 4 shows the estimated latency difference of the lowest RMSE resulting from the quadratic fit and of the bias of 0° resulting from the linear fit. Here, both estimated latency differences were very close together with a maximal distance of 1.6 ms for groups 1 and 2. For group 3, the best estimation of latency difference based on the bias was difficult because not all linear fit functions crossed zero close to the measured range. In these cases (S01, S04, S08, S12), the best latency difference is not shown.

The best latency difference between the value obtained directly from the linearly interpolated measured data and the value obtained from fitting the data differed by up to 2 ms in most subjects. In one case (bias of S11) it differed even more due to the highly non-monotonic data. These numbers match with our visual inspection, that best latency estimates have an uncertainty of 1–2 ms.

To obtain a single value for the best estimated latency difference, we have taken the mean of four values: the best estimated latency difference obtained from the RMSE and bias of the measured and fitted data (Figure 4, panel C). It is interesting to note that only for three subjects the estimated best latency difference was close to 0, which we define as the interval between −1 and 1 ms. This indicates that matching the latencies between the two ears is not a straightforward task.

Figure 4C also clearly shows that for group 1 the best localization performance was obtained for a positive estimated latency difference (faster CI), even though for S06 the best latency was close to 0 and, if based only on the RMSE data, the best latency was negative. For group 2, the best localization performance was obtained for a negative latency difference (faster hearing aid). Overall, most of our subjects seemed to localize best for Δτ values of 0 to 5 ms, that is, when $\tau_{e}\leq \tau_{a}$ , or equivalently when $\tau_{add}\leq \tau_{HA}$ .

**Figure 5.**
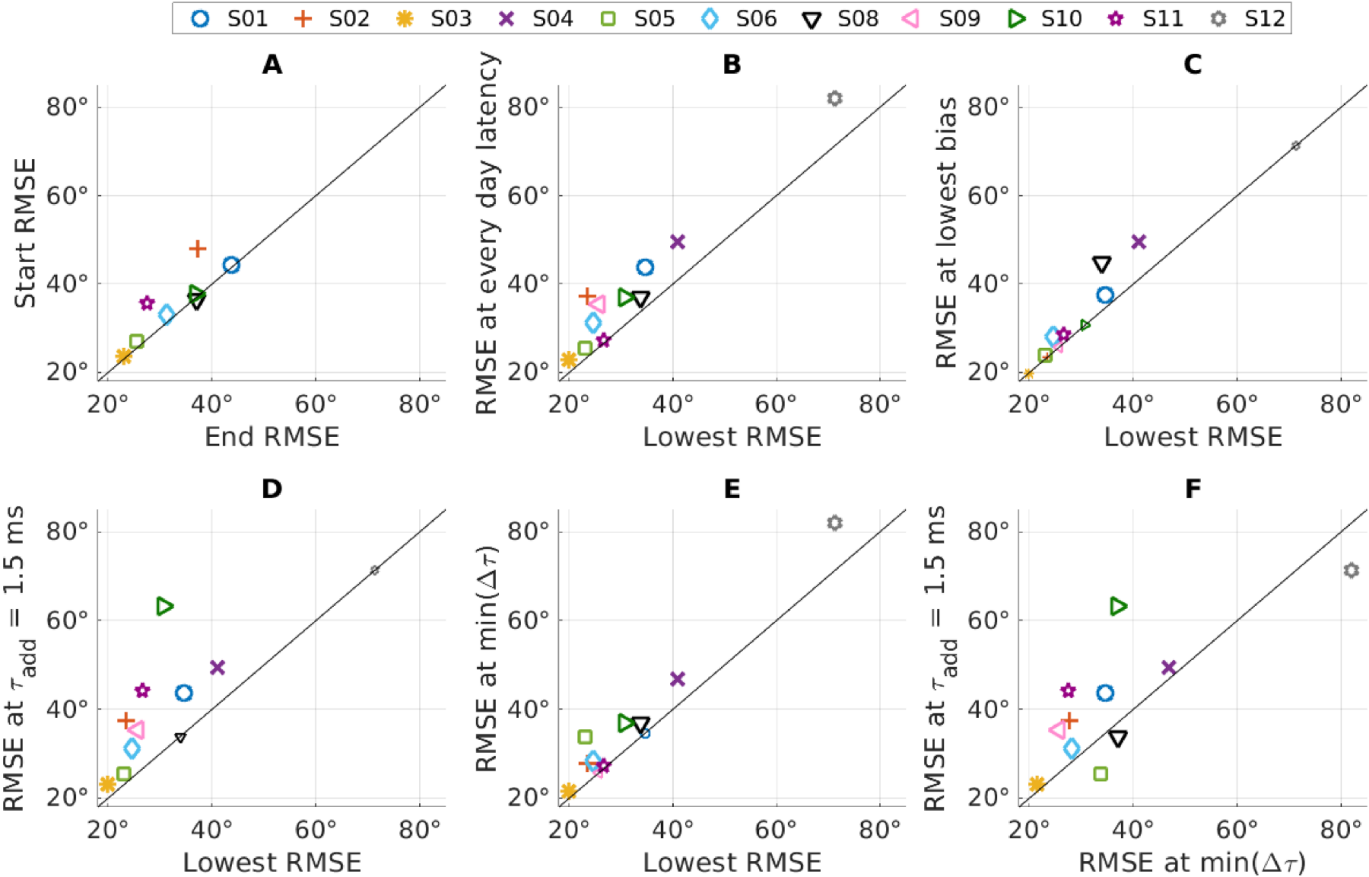
Pairwise Comparison of the Root Mean Square Error (RMSE) for Multiple Conditions. The Symbols Indicate the Data Points of the Individual Subjects. Values Above the Diagonal Indicate a Lower RMSE Value for the Condition on the *x*-Axis Compared to the Conditions on the *y*-Axis. A Higher Distance From the Diagonal Rates to a Higher Difference Between the Two Conditions. If the Values are the Same for Both Conditions, They Lie on the Diagonal and are Shown With Small Symbols.

In Figure 5, the RMSE values of the subjects for different conditions are compared pairwise. This was done by plotting the values in one condition against the values in the second condition. In this way, data points above the diagonal indicate larger RMSEs in conditions marked on the *y*-axis as compared to the condition on the *x*-axis. A greater distance from the diagonal indicates a greater difference between the two measures.

To evaluate the effect of training, we measured the same condition at the beginning and at the end (retest) of the measurement sessions for 8 of the 11 subjects. We used the latency settings that the subjects wore every day. The measured RMSE values of the start and end conditions can be seen in Figure 5, panel A. When comparing the values, only a small effect of training is visible (mean RMSE improvement: 3°) and most subjects were close to the diagonal (Figure 5A). However, as only one subject was slightly worse in the end condition and all other subjects improved their performance, an exact two-tailed Wilcoxon signed rank test showed a statistically significant difference (*p* = .02).

In the next step, we compared the RMSE measured with the everyday latency setting with the lowest measured RMSE of the respective participant obtained at any latency (Figure 5, panel B). For the eight subjects where a test-retest was done, the value of the retest measurement was used. The comparison showed a small improvement in the RMSE for every single subject, independent of the localization ability. The lowest RMSE is, per definition, always lower or equal to the value it is compared to. Therefore, a test for statistical difference would not be meaningful, as the difference would always be significant if not all values are equal. On average, the RMSE was reduced by 7° (17%) from 39° to 32°.

Following up on the comparison of the best latency estimated based on the lowest RMSE with the best latency estimated based on lowest bias (Figure 4), we now compare the corresponding RMSE values. For this, we plotted the RMSE at the lowest bias against the overall lowest RMSE in Figure 5, panel C. This comparison shows that even if the best latencies estimated based on bias or RMSE differed, the difference in RMSE for both conditions was on average only 3° (lowest RMSE: 32°, RMSE at lowest bias: 35°).

In the next step, we compared the RMSE measured with the lowest possible additional latency with the overall lowest measured RMSE (Figure 5, panel D). This central comparison demonstrates the total potential of latency optimization. With the present version of the programming software, the lowest additional latency is $\tau_{\mathrm{add}}=1.5\;$ ms. Only for S08 and the poorly localizing S12, the RMSE with $\tau_{\mathrm{add}}=1.5\;$ ms resulted in the lowest RMSE. The average improvement from the RMSE of the lowest additional latency to the lowest overall RMSE was 9° from 42° to 32°.

The simplest way to compensate for a latency difference in the clinical routine would be to set the additional latency equal to the hearing aid latency, as it was done by Angermeier et al. (2023). This results in an estimated latency difference Δτ=0 . Since we did not measure this value directly, we used the lowest estimated latency difference min(Δτ) instead. This value was between ±1 ms for all subjects. In Figure 5, panel E, we compare the RMSE at min(Δτ) with the overall lowest RMSE. The average improvement for all subjects from the RMSE at min(Δτ) to the overall lowest RMSE value was 4° from 37° to 32°.

Finally, we compared the RMSE of the lowest estimated latency difference (min(Δτ)) with the RMSE obtained when no additional latency was added ( $\tau_{\mathrm{add}}=1.5$ ms) (Figure 5, panel F). This shows that for most subjects a simple device latency-based compensation was beneficial, but for 3 of the 11 subjects the performance decreased. On average, the improvement in RMSE was 5°, but this was not statistically significant (exact two-tailed Wilcoxon signed rank test, *p* = .21).

## Discussion

In this study, we investigated the localization abilities of bimodal CI users with multiple latency differences between the CI and the hearing aid side. We have found that adjusting the additional CI latency immediately improved the localization ability of our bimodal CI subjects, which is consistent with previous studies of Angermeier et al. (2021, 2023).

The estimated latency difference that produced the best localization performance, both in terms of lowest RMSE and lowest absolute bias, was for most subjects not close to 0 ms estimated latency difference. There are several possible reasons for this. The first is that the latencies in both devices are frequency dependent. A frequency-independent compensation of the latency difference is certainly suboptimal, and the best value is likely to depend on both the dominant frequency band and on the frequency dependence of the hearing aid latency, which differs across manufacturers.

A second reason could be an adaptation to the latency difference. Although adaptation to the latency difference seems unlikely and the acute improvement in localization performance in our study and in the studies by Angermeier et al. (2021, 2023) argues against it, partial adaptation could play a role. None of the subjects in group 1 had a compensation for the latency difference in their clinical map, although all subjects had an estimated latency difference of at least 6 ms (Table 2). Looking at the RMSE over the estimated latency difference (Figure 3), it can be seen that all four subjects in this group benefited most from a CI faster than the hearing aid side. The theory of partial adaptation to a latency difference is also supported by the fact that both subjects in group 2 had a latency compensation in their clinical map and benefited the most from a CI slower than the hearing aid side. Interestingly, this was not observed in the data of Angermeier et al. (2021). In their study, most subjects preferred a slightly slower CI side even though none of them had a compensation for the latency mismatch. However, only additional CI latencies within ±1 ms of the estimated hearing aid latency were used by their measurements. Furthermore, their study was conducted with an older generation of the CI processor (Opus 2, here Sonnet 2), which might have had slightly different signal processing.

Another reason for the best localization performance at non-zero estimated latency difference could be an interaction between level mismatch and latency difference, as also suggested by Pieper et al. (2022). A level mismatch between the two ears causes an offset in the interaural level difference (ILD), which itself causes a localization bias. It is possible that this bias is partially compensated for by an opposite offset in ITD, that is, a latency difference (e.g., David et al., 1959; Stecker, 2010). This could also be the reason for the partial adaptation to the latency difference mentioned above. If the fitting procedures for both the hearing aid and the CI sides aim to achieve a balanced percept, this may result in partial compensation of the latency mismatch by a level difference. This partial compensation could then appear as an adaptation to the latency difference.

An interaction between the level mismatch and the latency difference can also be seen as an opportunity. A central goal of bimodal fitting is to achieve the best possible speech understanding for sounds from all directions. This might require an independent fitting of levels and gains on the two devices which can result in an interaural level mismatch. In other words, even when latency is compensated, there may be a level bias in sound localization, and compensating the level bias may reduce speech intelligibility. Our results suggest that the latency difference can be viewed not only as a problem but also as a degree of freedom, that can be used to partially compensate for a localization bias without compromising speech understanding in either ear.

If we compare the RMSE when no additional latency is used with the RMSE for a minimal latency difference, we found an improvement in the performance for 8 of our 11 subjects, similar to Angermeier et al. (2023). However, this improvement was not significant at the a group level in either our study or in Angermeier et al. (2023). In contrast, Angermeier et al. (2021) found a significant improvement in localization performance. A possible reason for this difference is that both in Angermeier et al. (2023) and in our work a minimal additional latency of 1.5 ms had to be used, due to the newer processor generation (Sonnet 2 instead of Opus 2). This means that the baselines used by Angermeier et al. (2023) and in our study differed from those in Angermeier et al. (2021) by an additional latency of 1.5 ms. Comparing the baseline performances, it can be seen that the initial RMSE also improved from 53° in Angermeier et al. (2021) to 40° in Angermeier et al. (2023), which showed that already the addition of the a small additional latency of 1.5 ms improved the localization performance.

Looking at the results of the individual subjects, it is interesting to note that both S08 and S12 had the lowest RMSE at an additional latency of 1.5 ms (Figure 5, panel D). The audiograms (Figure 1, panel B) showed that these two subjects still had good hearing thresholds in the low frequency range, which might allow for direct sound perception at those frequencies. If the low frequencies dominate the sound localization abilities, the best latency difference needs to be matched at the low frequencies. These two subjects illustrate once again that matching the latencies is not a straightforward task. It is highly dependent on the individual patient and not only on the hearing aid worn.

Both S08 and S12 are also two of the worst performing subjects. For S08, the bubble plots in Figure 2 show a pattern where everything was localized to the sides. This indicates an unfused sound perception. S12 perceived all sound sources as coming from the CI side. Although the RMSE was slightly lower for an additional latency of 1.5 ms, it was still much worse than the RMSE of any other subject in this study. Looking again at the audiogram, we can see that in addition to good low frequency hearing, S12 also had very bad high frequency hearing on the acoustic side. This might contribute to the advantage of the CI ear, especially since our stimuli were white noise bursts, which contain a higher amount of high frequency energy compared to most real-world stimuli.

Another subject with a rather poor localization performance is S04. Here, the non-monotonic change of the response with changing additional latency (see Figure 4) is particularly interesting. Multiple possible reasons arise when looking at the meta data. The first one is that S04 is by far the oldest of our subjects and at 86 years of age may have had some difficulty in performing this task. Additionally, four electrodes of the CI of S04 were deactivated during the fitting process due to incomplete insertion with at least two extra-cochlear electrodes. This shallower insertion likely caused a greater discrepancy in the characteristic frequency of the nerve fibers excited between the left and right ears. Note that variations in insertion depth are common (Hu & Dietz, 2015; Landsberger et al., 2015), to a degree that the tonotopic mismatch alone diminishes the processing of interaural cues (Bernstein et al., 2018; Hu & Dietz, 2015). Most critically in the present context, the combination of a tonotopic mismatch with a frequency-dependent device latency leads to an additional latency mismatch, when comparing latencies at matched tonotopic places (Williges et al., 2018). This tonotopically induced latency mismatch can easily reach 2 ms at low frequencies.

In the Introduction we hypothesized, in agreement with Pieper et al. (2022), that it should be possible to improve the localization abilities of bimodal CI users to a performance comparable to that of bilateral CI users. Therefore, we now compare the best possible localization results of the 11 subjects in our study with the localization performance of the study by Dorman et al. (2016). Just by finding the optimal latency difference for our subjects, a mean RMSE of 32° (median RMSE: 27°) can be achieved. This performance is very comparable to that of the bilateral CI users in the study by Dorman et al. (2016), who achieved a mean RMSE of 29° and a median of 27°. This shows that bimodal CI users are able to localize similarly to bilateral CI users when their devices latencies are optimally fitted.

The stimuli used in this study were Gaussian white noise burst triplets, as in Angermeier et al. (2021, 2023). They were used because in stimuli with several sharp onsets the latency difference or the ITD have a large influence on sound localization (Bernstein & Trahiotis, 2003; Dietz et al., 2015). However, stimuli with such sharp onsets are rare and in more natural stimuli with shallower envelopes, such as speech, the latency difference is less critical due to a reduced influence of the envelope ITD, as discussed by Pieper et al. (2022). It is therefore conceivable that a smaller benefit of the best latency would have been observed with more natural stimuli. It is even likely that some bimodal CI users have learned to localize (Trapeau & Schönwiesner, 2015) everyday-life sounds without a bias, even though there is a bias in their interaural cues and it is only the untrained prominent onset-ITDs that cause the bias in the present study. That said, it is not desirable that the majority of sounds are localized correctly, due to learned ILD-dominated spatial maps, but every now and then, a transient sound, which is often of ecological importance, is wrongly localized due to largely offset ITD cues. Furthermore, it is possible that stimuli with different spectral content lead to a different best latency difference, due to the frequency dependence of the latencies in the CI and the acoustic hearing side.

We do not expect that a latency mismatch compensation will directly improve in speech understanding in noise, due to the very limited benefit that even bilateral CI users get in the presumed absence of input asymmetries (e.g., de Graaff et al., 2021). This is in line with Angermeier et al. (2023) and Dolhopiatenko and Nogueira (2025), who did not find an improvement in speech perception in noise with a reduced latency mismatch. However, improved localization abilities are helpful in everyday hearing beyond speech-in-noise understanding. Also binaural fusion is facilitated by interaurally coherent input (Brown & Tollin, 2021). As the latest generation of MED-EL CIs allows to set an additional CI latency, it is recommended to take advantage of this opportunity to improve the hearing of the patients.

It is typically not feasible to perform the presented localization test during the clinical routine. As the difference between the lowest RMSE and the RMSE for the best bias (Figure 5, bottom left) is small, we suggest that minimizing bias can be used as a fitting goal for a clinical latency fitting routine. The bias could be estimated during the fitting by asking the patient from which direction (left, front, right) a sound was heard. The latency is then adjusted until a sound from the front is perceived as coming from the front. If patients are unable to perform this task, estimating the difference based on the hearing aid latency difference is also likely to improve the localization abilities.

## Conclusion

In our study, we measured the localization performance of 11 bimodal CI subjects for multiple latency differences between the two ears. We showed that adjusting the latency difference immediately improved localization performance for most subjects. Interestingly, the latency difference that produced the best localization performance was rarely 0 ms. Most subjects preferred a CI that was 2–5 ms faster than the estimated match, but two subjects performed best with a CI that was approximately 4 ms slower than estimated. We conclude that adjusting the latency difference is beneficial for binaural hearing. However, minimizing the latency difference is often not ideal and a dedicated test to find the optimal latency difference for the patient may be necessary. With the best possible latency difference for each subject, the average RMSE in our study was comparable to that of bilateral CI users in the study by Dorman et al. (2016). This shows that the bimodal CI users can achieve the same localization performance as bilateral CI users. Our results indicate that the latency difference can be considered as a degree of freedom, that allows to compensate for a localization bias due to a level mismatch between the two ears. We therefore recommend adjusting the latency difference as part of the clinical fitting routine. To facilitate this, it is desirable to develop a standardized, time-efficient clinical procedure that can reliably assess near-optimal latency.

## Notes

1.
https://www.medel.pro/online-resources/timing-settings-for-hearing-aids

2. The authors only became aware of the minimum latency of 1.5 ms to be set during the measurements. Therefore, the planned latencies were slightly adjusted after subject 08: 3 ms instead of 2 and 4.5 ms instead of 4.

## References

[bibr1-23312165251396658] Angermeier J. Hemmert W. Zirn S. (2021). Sound localization bias and error in bimodal listeners improve instantaneously when the device delay mismatch is reduced. Trends in Hearing, 25, 23312165211016165. 10.1177/23312165211016165 34057366 PMC8182625

[bibr2-23312165251396658] Angermeier J. Hemmert W. Zirn S. (2023). Clinical feasibility and familiarization effects of device delay mismatch compensation in bimodal CI/HA users. Trends in Hearing, 27, 23312165231171987. 10.1177/23312165231171987 37194477 PMC10196534

[bibr3-23312165251396658] Bernstein J. G. W. Stakhovskaya O. A. Schuchman G. I. Jensen K. K. Goupell M. J. (2018). Interaural time-difference discrimination as a measure of place of stimulation for cochlear-implant users with single-sided deafness. Trends in Hearing, 22, 2331216518765514. 10.1177/2331216518765514 29623771 PMC5894906

[bibr4-23312165251396658] Bernstein L. R. Trahiotis C. (2003). Enhancing interaural-delay-based extents of laterality at high frequencies by using “transposed stimuli”. The Journal of the Acoustical Society of America, 113(6), 3335–3347. 10.1121/1.1570431 12822805

[bibr5-23312165251396658] Brown A. D. Tollin D. J. (2021). Effects of interaural decoherence on sensitivity to interaural level differences across frequency. The Journal of the Acoustical Society of America, 149(6), 4630–4648. 10.1121/10.0005123 34241434 PMC8249038

[bibr6-23312165251396658] David E. E. Guttman N. van Bergeijk W. A. (1959). Binaural interaction of high-frequency complex stimuli. The Journal of the Acoustical Society of America, 31(6), 774–782. 10.1121/1.1907784

[bibr7-23312165251396658] de Graaff F. Eikelboom R. H. Sucher C. Kramer S. E. Smits C. (2021). Binaural summation, binaural unmasking and fluctuating masker benefit in bimodal and bilateral adult cochlear implant users. Cochlear Implants International, 22(5), 245–256. 10.1080/14670100.2021.1894686 33832408

[bibr8-23312165251396658] Dietz M. Klein-Hennig M. Hohmann V. (2015). The influence of pause, attack, and decay duration of the ongoing envelope on sound lateralization. The Journal of the Acoustical Society of America, 137(2), EL137–EL143. 10.1121/1.4905891 25698041

[bibr9-23312165251396658] Dolhopiatenko H. Nogueira W. (2025). Cortical temporal mismatch compensation in bimodal cochlear implant users: Selective attention decoding and pupillometry study. Hearing Research, 464, 109306. 10.1016/j.heares.2025.109306 40412302

[bibr10-23312165251396658] Dorman M. F. Loiselle L. H. Cook S. J. Yost W. A. Gifford R. H. (2016). Sound source localization by normal-hearing listeners, hearing-impaired listeners and cochlear implant listeners. Audiology & Neurotology, 21(3), 127–131. 10.1159/000444740 27077663 PMC4949120

[bibr11-23312165251396658] Engler M. Digeser F. Hoppe U. (2020). Bestimmung interauraler Zeitdifferenzen bei bimodaler Versorgung. Deutsche Gesellschaft für Audiologie e.V.

[bibr12-23312165251396658] Hartmann W. M. Macaulay E. J. (2014). Anatomical limits on interaural time differences: An ecological perspective. Frontiers in Neuroscience, 8, 34. 10.3389/fnins.2014.00034 24592209 PMC3937989

[bibr13-23312165251396658] Hu H. Dietz M. (2015). Comparison of interaural electrode pairing methods for bilateral cochlear implants. Trends in Hearing, 19, 10.1177/2331216515617143 PMC4771032 26631108

[bibr14-23312165251396658] Landsberger D. M. Svrakic M. Roland J. T. Svirsky M. (2015). The relationship between insertion angles, default frequency allocations, and spiral ganglion place pitch in cochlear implants. Ear and Hearing, 36(5), e207–e213. 10.1097/AUD.0000000000000163 PMC4549170 25860624

[bibr15-23312165251396658] Mills A. W. (1958). On the minimum audible angle. The Journal of the Acoustical Society of America, 30(4), 237–246. 10.1121/1.1909553

[bibr16-23312165251396658] Pieper S. H. Hamze N. Brill S. Hochmuth S. Exter M. Polak M. Radeloff A. Buschermöhle M. Dietz M. (2022). Considerations for fitting cochlear implants bimodally and to the single-sided deaf. Trends in Hearing, 26, 23312165221108259. 10.1177/23312165221108259 35726211 PMC9218456

[bibr17-23312165251396658] Ruggero M. A. Temchin A. N. (2007). Similarity of traveling-wave delays in the hearing organs of humans and other tetrapods. Journal of the Association for Research in Otolaryngology : JARO, 8(2), 153–166. 10.1007/s10162-007-0081-z 17401604 PMC1868567

[bibr18-23312165251396658] Seebacher J. Franke-Trieger A. Weichbold V. Zorowka P. Stephan K. (2019). Improved interaural timing of acoustic nerve stimulation affects sound localization in single-sided deaf cochlear implant users. Hearing Research, 371, 19–27. 10.1016/j.heares.2018.10.015 30439571

[bibr19-23312165251396658] Stecker G. C. (2010). Trading of interaural differences in high-rate Gabor click trains. Hearing Research, 268(1-2), 202–212. 10.1016/j.heares.2010.06.002 20547218 PMC2923247

[bibr20-23312165251396658] Trapeau R. Schönwiesner M. (2015). Adaptation to shifted interaural time differences changes encoding of sound location in human auditory cortex. NeuroImage, 118, 26–38. 10.1016/j.neuroimage.2015.06.006 26054873

[bibr21-23312165251396658] Williges B. Jürgens T. Hu H. Dietz M. (2018). Coherent coding of enhanced interaural cues improves sound localization in noise with bilateral cochlear implants. Trends in Hearing, 22, 2331216518781746. 10.1177/2331216518781746 29956589 PMC6048749

[bibr22-23312165251396658] Zirn S. Arndt S. Aschendorff A. Wesarg T. (2015). Interaural stimulation timing in single sided deaf cochlear implant users. Hearing Research, 328, 148–156. 10.1016/j.heares.2015.08.010 26302945

[bibr23-23312165251396658] Zirn S. Müller F-U Roth S. Angermeier J. Hemmert W. (2025). The effect of broadband and frequency-specific delay compensation in bimodal CI users. In Conference on Implantable Auditory Prostheses.

